# From the Editor’s Desk

**Published:** 2023

**Authors:** Pat Commerford

**Figure:**
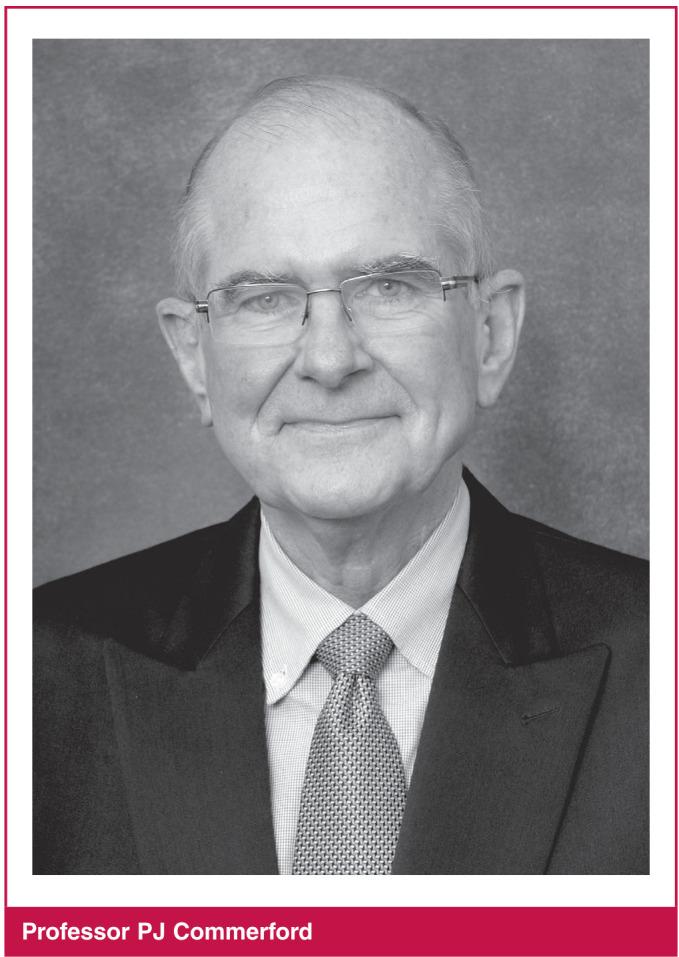


It is often difficult to understand the reluctance of some medical practitioners to accept generic substitutes of the originator medicine. Of course, there are seldom large-scale randomised clinical trials to prove equivalence but most reputable regulatory authorities register generics only after careful evaluation. Given the cost-saving consequences of generic substitution, it is understandable that most publicly funded health services require generic substitution in an attempt to spread the benefit to a larger population. To my knowledge there has not been any solid scientific proof that such substitution is in any way harmful. In this final issue of 2023, Snyman and colleagues (page 264) describe a retrospective, chart-based review of the effects of switching patients with hyperlipdaemia from the originator statin to a generic. They concluded this real-world evidence should allay any fears of generic inferiority of this important medicine in the treatment and prevention of high cardiovascular risk in patients requiring lipid-lowering therapy. The study was retrospective and chart-based but given those limitations, the results should be reassuring to those practicing in resource-poor settings.

 In a similar vein, Acheampong and colleagues from Ghana, collaborating with those in the USA (page 268) describe the outcome of an important evaluation, not of treatment, but of the investigation of patients with cardiovascular disease by training non-cardiologists in echocardiography performance and interpretation. Internal medicine residents at a tertiarylevel hospital in Ghana were trained to perform limited echocardiographic studies. Interpretation was compared to expert interpretation. Agreement improved over time. At the final evaluation, there was high agreement across all aspects: left ventricular structure and function, right ventricular structure and function, and presence of effusion. This accords with general experience. From the very early days of M-mode echocardiography, non-cardiologists and clinical technologists have been trained to perform these relatively simple measures successfully and it is reassuring to have it quantified and confirmed in a formal investigation. Certainly, evaluations of more complex aspects will require much more extensive training.

 Many consider that management of hypertension in Africa leaves much to be desired. A systematic review of the subject by Cavagna and co-authors (page 307) confirms this opinion.

 Budhram and Krishundutt (page 285) report a three-year audit of pregnancy outcomes in women with pulmonary hypertension admitted to a high-risk obstetric unit in South Africa. An important letter to the editor by Ilonze (page 291) points out that the results may not necessarily be generalisable because of both the method of diagnosing pulmonary hypertension (echocardiography) and the characteristics of the patient population. Such detailed critique is welcomed and will always be published. 

